# Long noncoding RNA hypoxia-inducible factor 1 alpha-antisense RNA 1 promotes tumor necrosis factor-α-induced apoptosis through caspase 3 in Kupffer cells

**DOI:** 10.1097/MD.0000000000009483

**Published:** 2018-01-26

**Authors:** Yanghe Wu, Jiguang Ding, Qingfeng Sun, Ke Zhou, Weiwei Zhang, Qingwei Du, Tingyan Xu, Wangwang Xu

**Affiliations:** Infectious Disease, Ruian People's Hospital, Ruian, Zhejiang Province, P.R. China.

**Keywords:** apoptosis, HIF1A-AS1, Kupffer cells, TNF-α

## Abstract

Kupffer cells (KCs) play a crucial role in the pathogenesis of acute-on-chronic liver failure (ACLF) which is characterized by acute and severe disease in patients with preexisting liver disease and shows high mortality. Long noncoding RNAs (lncRNAs) are recently found to be involved in gene regulation. However, the mechanisms of how KCs are regulated by inflammatory factors, tumor necrosis factor-α (TNF-α), and whether lncRNAs are involved in the process remain largely unknown. Hence, we investigated the role of lncRNAs in the cytotoxicity of TNF-α on KCs.

lncRNA array (The lncRNAs in the array are apoptosis-related lncRNAs reported in some research papers.) was used to identify lncRNAs related with liver fibrosis. Annexin V/protease inhibitor (PI) staining was used for detection of cell apoptosis. Real time-polymerase chain reaction was utilized for analysis of mRNA levels of lncRNA hypoxia-inducible factor 1 alpha-antisense RNA 1 (HIF1A-AS1) and apoptosis-related genes. Western blot was implied to the determination of lymphoid enhancer factor-1 (LEF-1).

In this study, we found that HIF1A-AS1 could be upregulated by TNF-α by lncRNA array analysis and knockdown of HIF1A-AS1 significantly rescued cell apoptosis induced by TNF-α. Moreover, inhibition of HIF1A-AS1 markedly reduced mRNA level of caspase 3 which can be significantly enhanced by TNF-α. Furthermore, HIF1A-AS1 showed binding sites for LEF-1 and siRNA-mediated downregulation of LEF-1 decreased HIF1A-AS1 level in KCs treated with TNF-α.

This study elucidates a new role of HIF1A-AS1 in TNF-α-induced cell apoptosis and provides potential therapeutic targets for ACLF.

## 1 Introduction

Acute-on-chronic liver failure (ACLF) is a clinical syndrome of acute and severe hepatic dysfunction on an underlying chronic liver disease with high 28-day mortality.^[1]^ It is often induced by alcohol, hepatotropic viruses, and drugs whereas the previous chronic liver disease is often due to factors, such as alcohol, hepatitis B or C, or non-alcoholic steatohepatitis (NASH).^[1]^ The major pathological changes are necrosis of liver cells, infiltration of immune cells, and release of inflammatory cytokines.^[2–4]^ Although with high mortality and poor prognosis, the accurate biological mechanisms for ACLF are still lacking. Therefore, understanding the potential pathogenesis of ACLF will facilitate the recognition, treatment, and prognostic therapy.

Kupffer cells (KCs) are resident macrophages in the liver that are at the first line to defense for pathogens derived from gut.^[5]^ Kupffer cells can be activated through different mechanisms, such as lipopolysaccharide (LPS), TLR4, complement C3 or C5 signaling pathways, when the liver encounter acute injury or the intestine shows increased permeability.^[5]^ At early stage of ACLF, KCs release tumor necrosis factor (TNF) and interleukin-6 (IL-6), while at late stage, KCs mainly releases anti-inflammatory cytokine, IL-10.^[4]^ In addition, in response to HBV activation, peripheral blood mononuclear cell (PBMC) can release many pro-inflammatory cytokines, which include IL-6, IL-8, IL-10, IL-12, p70, IL-1β, and tumor necrosis factor-α (TNF-α).^[6]^ Of these cytokines, TNF-α is especially crucial in the pathogenesis of liver dysfunction and its plasma level is well correlated with the severity of liver injury and development of soluble immune response suppressor (SIRS).^[7–9]^ However, the mechanism by which TNF-α activates KCs remains to be clarified.

Long noncoding RNAs (lncRNAs) are generally defined as endogenous cellular RNAs with length longer than 200 nucleotides but with no protein coding potential, which are pervasively found in eukaryotic genomes.^[10]^ Based on their location to nearby genes, lncRNAs can be divided into different groups: sense, antisense, intergenic, and bidirectional lncRNAs.^[11]^ Evidences have shown that lncRNAs play essential roles in maintaining cellular homeostasis during cell or tissue development and even critical for tumorgenesis.^[12]^ In addition, lncRNAs are also important for cell proliferation, cell apoptosis, chromatin regulation, and cell-cycle progression.^[11]^ Whether lncRNAs play a role in TNF-α-induced apoptosis in KCs remains largely unknown. Elucidating the biological mechanisms of how lncRNAs work in that process will provide new insight into the clinical application of KCs in ACLF.

In the present study, we identified HIF 1 alpha-antisense RNA 1 (HIF1A-AS1) through high-throughput screening, and elucidated its proapoptotic role in TNF-α-induced apoptosis in KCs. Mechanistically, HIF1A-AS1 enhanced TNF-α-induced apoptosis by upregulating the expression caspase 3. Our data suggested that lncRNA HIF1A-AS1 might serve as a therapeutic target for ACLF patients.

## 2 Methods

### 2.1 Ethical approval

Ethical approval was not necessary in the paper due to that our work focused on mechanism research of TNF-α on Kupffer cells and the study did not involved clinical patients or animal experiments.

### 2.2 Cell culture

KCs were cultured in RPMI1640 medium supplemented with 10% fetal bovine serum (FBS; HyClone) and 1% penicillin/streptomycin (Pen/Strep) in a 5% CO_2_ humiditified atmosphere at 37 °C.

### 2.3 Halfmaximal inhibitory concentration (IC_50_) detection

The apoptosis-inducing agent TNF-α was used to determine the cell viability with 3-(4, 5-dimethylthiazol-2-yl)-2, 5-diphenyltetrazolium bromide Methylthiazolyldiphenyl-tetrazolium bromide (MTT) assay, as described elsewhere.^[13]^ In brief, KCs in the logarithmic phase of growth were stained with trypan blue to count the viable cell under an inverted phase contrast microscope. Then the cell density was adjusted to 1 × 10^5^ cells/mL. Hundred microliter of the cell suspension was plated into a 96-well culture plate. TNF-α were added into the cells at final concentrations of 0 (control group), 2.5, 5, 7.5, 10, 12.5 ng/mL, and cells were cultured with TNF-α for 48 hours. Four hours before end of culture, MTT was added into the culture medium. After incubation for 4 hours, cells were centrifuged at 153 × *g* for 5 minutes and supernatant were decanted. Crystals were fully dissolved with 150 μL dimethyl sulfoxide for 15 minutes at room temperature. The absorbance was measured with a spectrophotometer at 560 nm. The viability rate was calculated as follows: cell viability (%) = [optical density (OD) of the test/OD of the control] × 100%. The IC_50_ was determined with LOGIT method.

### 2.4 Analysis of apoptosis by flow cytometry

Cell apoptosis was analyzed with Annexin V/PI apoptosis detection kit (BD, CA) according to the manufacturer's instructions. Briefly, 2.5 × 10^5^ cells were plated into 6-well plates and were subjected to different treatment. Forty-eight hours later, cells were digested with accutase for 1 to 2 minutes at room temperature. Two milliliter of medium was added to each well and the contents (∼3 mL) were transferred to the 15-mL tubes. Then, cells were centrifuged and the supernatant was discarded. After washed with ice-cold PBS for twice, cells were suspended in 1 time Binding Buffer at a concentration of ∼1 × 10^6^ cells/mL. Hundred microliter of the solution was used for reaction, and 5 μL of fluorescein isothiocyanate (FITC)-Annexin V was added. The tubes were incubated for 30 minutes in dark at room temperature (RT). Then, PI was added and reaction was performed at dark for 5 minutes. Finally, 400 μL of 1 time Binding Buffer was added to each tube and cells were analyzed by flow cytometry as soon as possible (within 1 hour). Unstained cells, cells stained with FITC-Annexin V (no PI) and cells stained with PI (no FITC-Annexin V) were for compensation set up.

### 2.5 Real-time PCR

Total RNA was extracted from KCs using Trizol Reagent (Invitrogen, CA) according to the instructions. RNA was quantified with a NanoDrop ND-1000 spectrophotometer (organization-Thermo Fisher Scientific Inc, Waltham, MA) at OD 260 nm. The quality of RNA was assessed with standard denaturing agarose gel electrophoresis and purity was determined with the absorbance at 260 and 280 nm. cDNA was synthesized with 2 μg of total RNA. The PCR reaction was performed in a volume of 20 μL and done for triplicates (PrimeScript RT reagent Kit with gDNA Eraser, Takara, RR047A). The cycling conditions for the synthesis of PCR were initial denaturation at 95 °C for 1 minute, denaturation at 95 °C for 15 seconds, annealing at 60 °C for 20 seconds, extension at 72 °C for 40 seconds (40 cycles), and a final extension step at 72 °C for 7 minutes (SYBR Premix Ex Taq II [Tli RNaseH Plus], ROX plus, Takara, RR82LR). Housekeeping glceraldehyde-3-phosphatedehydrogenase mRNA was used as internal control for comparison of relative RNA expressions. Relative gene expression was evaluated using the 2^(−ΔΔCT)^ method.^[14]^ (The information of primers is Table 1 in Support Materials.)

**Table 1:**
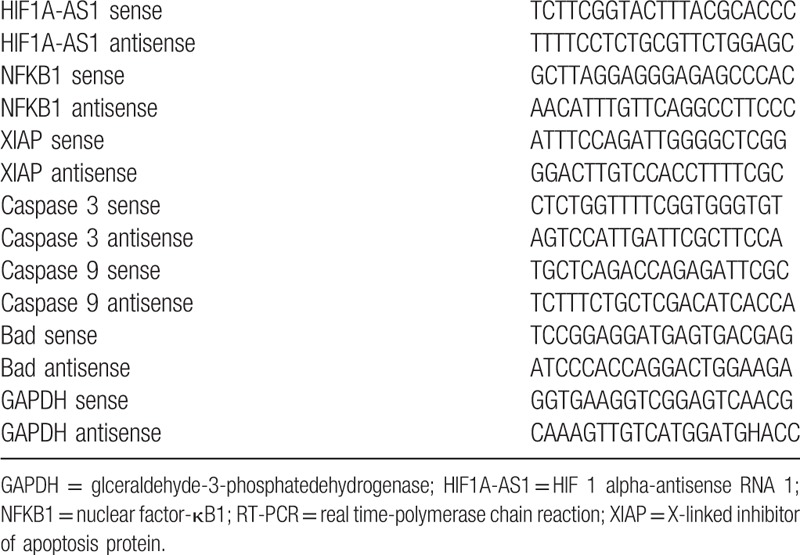
RT-PCR primers in the paper.

### 2.6 Western blot

Total protein was extracted from KCs in ice-cold lysis buffer (50 mmol/L Tris–HCl pH 7.5, 150 mmol/L NaCl, 5 mmol/L EDTA, 1% NP-40) containing a PI cocktail (Roche). Equal protein was fractionated by 12% polyacrylamide gel electrophoresis and electrophoretically transferred to polyvinylidene difluoride membranes. The membranes were blocked with 5% non-fat milk for 2 hours at room temperature, and then incubated with primary antibodies (Anti-LEF1 antibody: abcam, ab52017, dilution: 1 μg/mL; Anti-β-Actin antibody: abcam, ab8226, dilution: 1:5000) at 4 °C overnight. After washing with PBST for 3 times, the membranes were subjected to indicated secondary antibodies (Goat anti-Rat IgG H&L [FITC] secondary antibody, abcam, ab6840, dilution: 1:5000; Goat Anti-Rabbit IgG H&L [FITC], abcam, ab6717, dilution: 1:5000) for 2 hours at room temperature. The bands were further visualized with enhanced chemiluminescence (Pierce) by LAS 4000.^[15]^

### 2.7 Statistical analysis

Statistical analysis was done using SPSS version 13.0 for Windows (SPSS, Inc., Chicago, IL). All data were expressed as means ± SEM. Differences between groups were determined with Student's *t* test. *P* value <.05 was considered to indicate a statistically significant difference. The *P* value were marked in the figures by using asterisks, and indicated in the figure legends as ^∗^: *P* < .05, ^∗∗^: *P* < .01, ^∗∗∗^: *P* < .001.

## 3 Results

### 3.1 TNF-α induced Kupffer cell apoptosis

**Figure 1:**
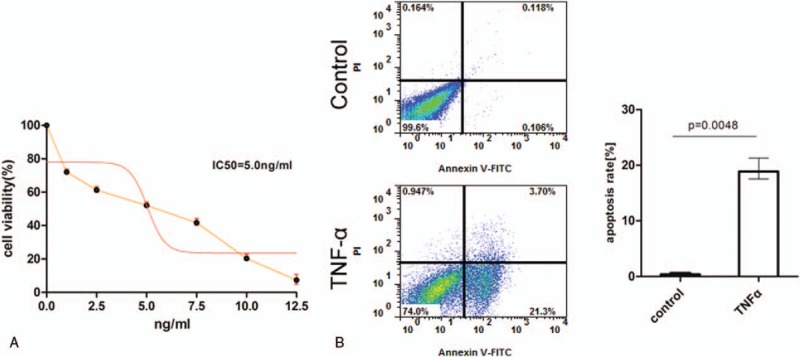
Effect of TNF-α on KCs apoptosis. (A) KCs treated with different concentrations (0, 2.5, 5, 7.5, 10, 12.5 ng/mL) of TNF-α for 48 hours were analyzed by MTT assay. The percentage of viable cells is shown. (B) Representative flow cytometry plots (left panel) and statistical analysis (right panel) of KCs stained with Annexin V and PI to examine the effect of TNF-α on KCs apoptosis. KCs were treated without or with 5 ng/mL of TNF-α and were detected 24 hours later. KCs = Kupffer cells; MTT = Methylthiazolyldiphenyl-tetrazolium bromide; PI = protease inhibitor; TNF-α = tumor necrosis factor-α.

In order to detect the effect of TNF-α on KCs, MTT assay was performed on KCs treated with different concentrations of TNF-α for 48 hours. The results showed that as with the increase of TNF-α concentration, KCs showed decreased cell viability, revealing a dose-dependent manner (Fig. 1A). In addition, the mean IC_50_ value of TNF-α was 5 ng/mL (Fig. 1A). Next, we investigated whether TNF-α could induce KCs apoptosis by using Annexin V/PI staining and flow cytometry. As shown in Fig. 1B, KCs showed significant apoptosis (21.3%) when they were treated with TNF-α for 24 hours at a concentration of 5 ng/mL. These data suggested that TNF-α inhibited KCs viability in a dose-dependent manner and induced KCs apoptosis.

### 3.2 TNF-α increased the expression of HIF1A-AS1

**Figure 2:**
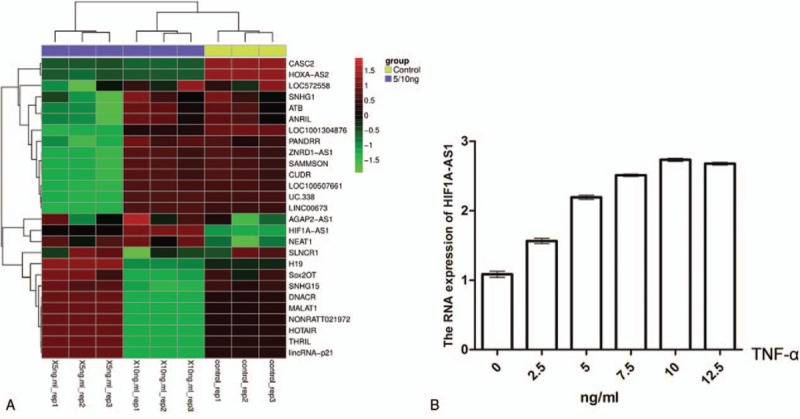
Effect of TNF-α treatment on the expressions of lncRNAs in KCs. (A) Heat map showing hierarchical clustering of lncRNAs with differential expression at FDR < 0.05 and *P* < .001 in KCs treated without or with 5 and 10 ng/mL of TNF-α. Green represents a decrease in expression level, whereas red represents an increase. FDR was for false-discovery rate. (B) Representative real-time PCR data showing the mRNA levels of lncRNA HIF1A-AS1 in KCs treated for 24 hours with TNF-α at concentrations of 0, 2.5, 5, 7.5, 10, 12.5 ng/mL. The expressions of HIF1A-AS1 were normalized to internal control GAPDH. GAPDH = glceraldehyde-3-phosphatedehydrogenase; KCs = Kupffer cells; lncRNA = long noncoding RNA; PCR = polymerase chain reaction; TNF-α = tumor necrosis factor-α.

To identify the potential target of TNF-α in the process of apoptosis, we predicated several apoptosis-related lncRNAs (The lncRNAs in the array are apoptosis-related lncRNAs reported in some research papers^[16–41]^) or genes and checked their expressions during apoptosis of KCs. Heatmap visualization of known apoptosis markers demonstrated that HIF1A-AS1 was one of the significantly upregulated lncRNAs when KCs were activated by TNF-α (Fig. 2A). Furthermore, we confirmed HIF1A-AS1 expression in KCs in response to TNF-α treatment by real-time PCR. Results showed that HIF1A-AS1 expression was increased in a TNF-α dose-dependent manner (Fig. 2B). Taken together, these results demonstrated that TNF-α enhanced expression of HIF1A-AS1 in KCs.

### 3.3 Downregulation of HIF1A-AS1 reduced TNF-α-induced apoptosis in KCs

**Figure 3:**
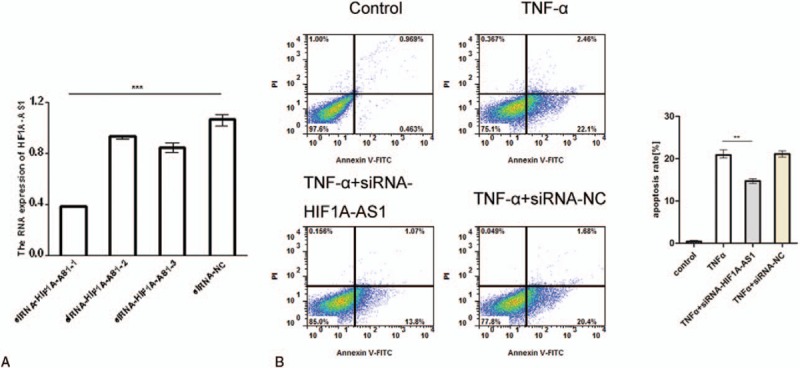
Effect of knockdown of HIF1A-AS1 on TNF-α induced KCs apoptosis. (A) Quantitative analysis of HIF1A-AS1 levels in KCs which were transfected with different HIF1A-AS1 siRNAs or siRNA control. Real-time PCR was performed 24 hours after transfection. ^∗∗∗^*P* < .001. (B) KCs were transfected with HIF1A-AS1 siRNAs or siRNA control, and simultaneously treated for 24 hours without or with 5 ng/mL TNF-α. Flow cytometry plots showing KCs apoptosis under different conditions detected by Annexin V and PI staining (left panel). Statistical analysis of 3 independent experiments was shown (right panel). HIF1A-AS1 = HIF 1 alpha-antisense RNA 1; KCs = Kupffer cells; PCR = polymerase chain reaction; PI = protease inhibitor; TNF-α = tumor necrosis factor-α.

TNF-α induced cell apoptosis and selectively increased the expression level of HIF1A-AS1, which lead us to raise the hypothesis that HIF1A-AS1 might be a key mediator in the process of TNF-α-induced apoptosis in KCs. To test this, we first designed 4 different pairs of siRNAs (Table 2 in Support Materials) and transfected them into KCs. The knockdown efficacy was validated by real-time PCR and si-RNA-HIF1A-AS1-1 could most significantly downregulate HIF1A-AS1 expression when compared with the control siRNA (Fig. 3A). We observed the effect of HIF1A-AS1 knockdown on KCs apoptosis after TNF-α treatment. As expected, when HIF1A-AS1 expression was downregulated in KCs that were treated with TNF-α, apoptosis percentage was significantly lower compared with KCs transfected with control siRNA and treated with TNF-α (Fig. 3B). These results revealed a contributing role of HIF1A-AS1 in the pro-apoptotic effect of TNF-α in KCs.

**Table 2:**
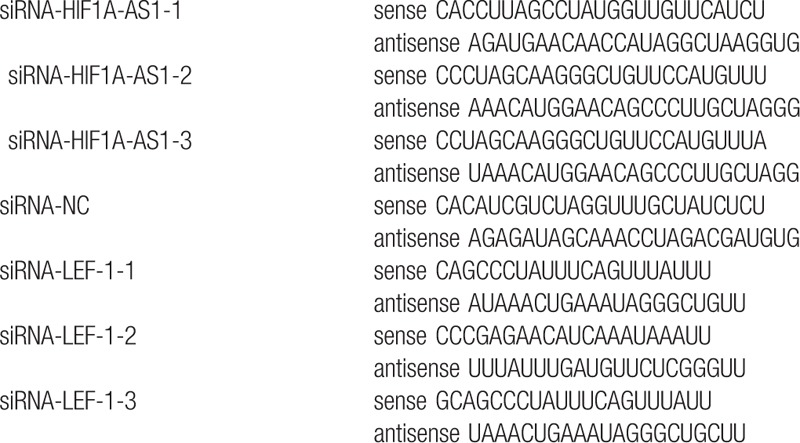
siRNAs in the paper.

### 3.4 HIF1A-AS1 promoted TNF-α-induced apoptosis by increasing caspase 3 expression in KCs

**Figure 4:**
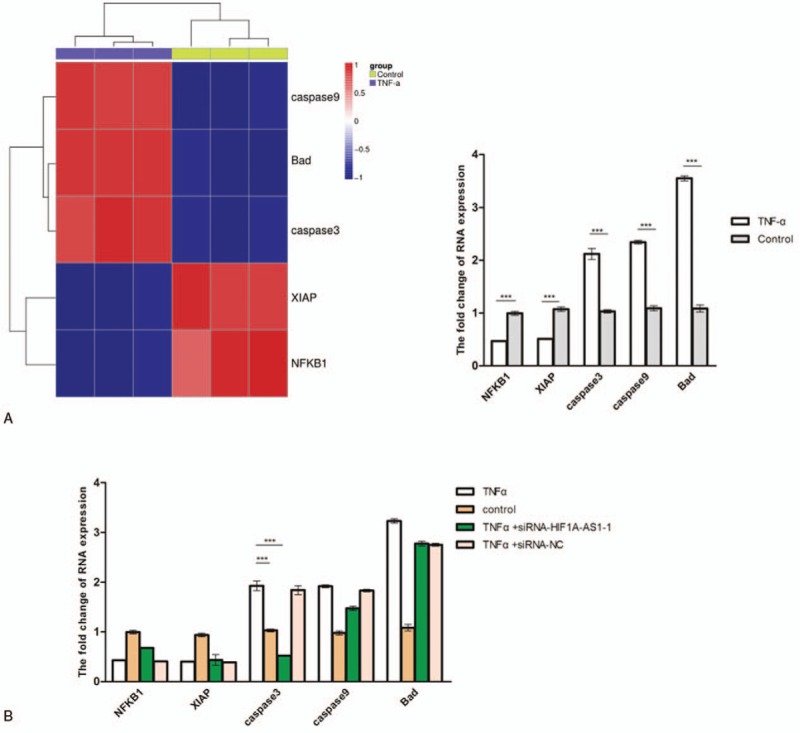
Role of HIF1A-AS1 in TNF-α-mediated apoptotic pathway. (A) Heat map for identification of TNF-α target genes by hierarchical clustering. The apoptosis-related genes were detected 24 hours after KCs treated without or with 5 ng/mL of TNF-α. Upregulated and downregulated genes are shown by red and green, respectively (left panel). Validation of the mRNA levels of NFKB1, XIAP, caspase 3, caspase 9, or Bad is shown by real-time PCR (right panel). KCs were treated as previously indicated. ^∗∗∗^*P* < .001. (B) Relative mRNA levels of indicated genes were detected in KCs that were transfected with control or siRNAs for HIF1A-AS1 and then treated with no or 5 ng/mL of TNF-α for 24 hours. Expression levels of indicated genes were normalized to that of GAPDH. ^∗∗∗^*P* < .001. GAPDH = glceraldehyde-3-phosphatedehydrogenase; HIF1A-AS1 = HIF 1 alpha-antisense RNA 1; KCs = Kupffer cells; NFKB1 = nuclear factor-κB1; PCR = polymerase chain reaction; TNF-α = tumor necrosis factor-α; XIAP = X-linked inhibitor of apoptosis protein.

To elucidate apoptotic pathways that were involved in TNF-α-treated KCs, we detected 5 apoptotic markers, which were nuclear factor-κB1 (NFKB1), X-linked inhibitor of apoptosis protein (XIAP), caspase 3, Bad, and caspase 9. Interestingly, real-time PCR analysis demonstrated that TNF-α induced an approximately 2-fold decrease of NFKB1, XIAP, but 2-fold increase of caspase 3, caspase 9, and about 3-fold increase of Bad in KCs when compared with the control cells (Fig. 4A). Furthermore, to investigate which genes that HIF1A-AS1 targeted in TNF-α-associated apoptotic pathways, KCs were transfected with siRNA-HIF1A-AS1-1 and control siRNA. Our results showed that the expression levels of NFKB1, XIAP, caspase 9, and Bad were not significantly changed by the downregulation of HIF1A-AS1 in KCs treated with TNF-α in comparison of KCs transfected with control siRNA and treated with TNF-α (Fig. 4B). However, HIF1A-AS1 downregulation significantly rescued the increase of caspase 3 which was led by TNF-α treatment (Fig. 4B). These data suggested that HIF1A-AS1 enhanced KCs apoptosis induced by TNF-α, at least for a part, by upregulating the expression of caspase 3.

### 3.5 TNF-α enhanced HIF1A-AS1 expression level through LEF-1

**Figure 5:**
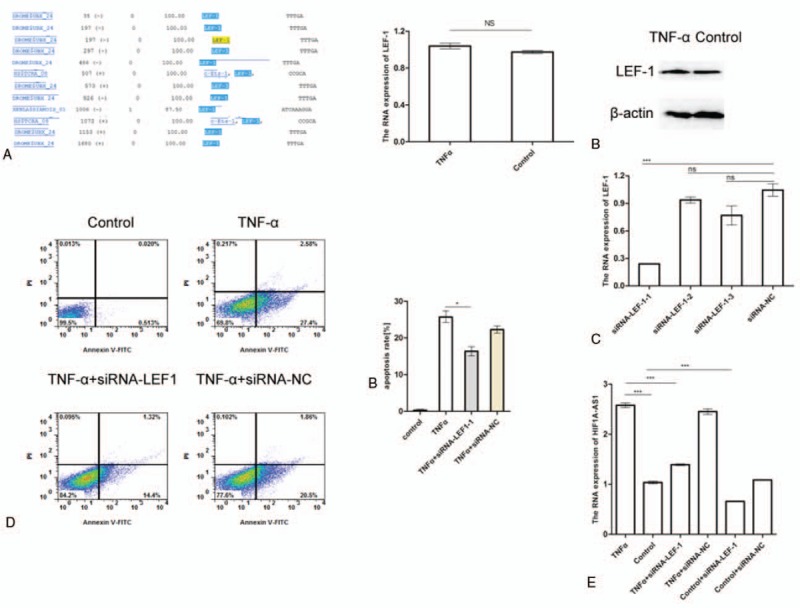
Identification of LEF-1 as transcription factor for HIF1A-AS1 in TNF-α-induced apoptosis. (A) Potential binding sites for LEF-1 in the promoter region of HIF1A-AS1 were predicated at http://www.gene-regulation.com/pub/programs.html (left panel). mRNA levels of LEF-1 were examined by real-time PCR in KCs treated with no or 5 ng/mL TNF-α for 24 hours (right panel). (B) Western blot analysis shows the protein levels of LEF-1 in KCs treated with no or 5 ng/mL TNF-α for 24 hours. β-action was used for internal control. (C) Identification of the knockdown efficiency of LEF-1 by real-time PCR. KCs were treated with siRNAs for LEF-1 and siRNAs control for 24 hours, and were used for mRNA level detection. (D) KCs were transfected with LEF-1 siRNAs or siRNA control, and then treated for 24 hours without or with 5 ng/mL TNF-α. Flow cytometry plots showing KCs apoptosis under different conditions detected by Annexin V and PI staining (left panel). Pool data from 3 independent experiments were analyzed (right panel). (E) The HIF1A-AS1 expression was detected by real-time PCR in KCs transfected with control or LEF-1 siRNAs treated with or without 5 ng/mL TNF-α for 24 hours. ^∗∗∗^*P* < .001. HIF1A-AS1 = HIF 1 alpha-antisense RNA 1; KCs = Kupffer cells; ns = not significant; PCR = polymerase chain reaction; PI = protease inhibitor; TNF-α = tumor necrosis factor-α.

How TNF-α regulated the expression of HIF1A-AS1 remained to be investigated. Thus, we predicated the binding sites for transcription factors in the promoter region of HIF1A-AS1. We found 11 binding sites for lymphoid enhancer factor-1 (LEF-1) (Fig. 5A), suggesting that LEF-1 might regulate HIF1A-AS1 expression. Next, the mRNA and protein levels of LEF-1 were examined. As shown in Fig. 5A and B, no significant increase of LEF-1 level was observed in KCs after treatment with TNF-α. Additionally, the effect of LEF-1 on the apoptotic role of TNF-α was investigated. The expression level of LEF-1 detected by real-time PCR was decreased by siRNA transfection in KCs (Fig. 5C). Then the effect of LEF-1 in cell apoptosis was detected in KCs which were treated with control, TNF-α, TNF-α+ siRNA-LEF-1, and TNF-α+ siRNA-NC. Importantly, when LEF-1 was knocked down using siRNAs, the percentage of TNF-α-induced apoptosis was significantly decreased (Fig. 5D), implying a proapoptotic role of LEF-1. Taking the facts into consideration that HIF1A-AS1 showed LEF-1 binding sites and LEF-1 promoted the apoptotic effect of TNF-α, it's reasonable for us to speculate that LEF-1 might regulate the expression of HIF1A-AS1. To verify this, we evaluated the mRNA levels of HIF1A-AS1 in KCs treated with TNF-α and where LEF-1 expression was knocked down. Results showed that the expression of HIF1A-AS1 was decreased in KCs treated with TNF-α+ si RNA-LEF-1 in compared with those treated with TNF-α or treated with TNF-α+ si RNA-NC (Fig. 5E). From the data above, it can be concluded that KCs with lower expression of LEF-1 exhibited lower expression of HIF1A-AS1 after TNF-α treatment, suggesting LEF-1 might serve as a mediator that TNF-α regulated HIF1A-AS1.

## 4 Discussion

The present study demonstrated that TNF-α could induce cell apoptosis of Kupffer cells in a dose-dependent manner. We further found that TNF-α significantly increased the expression level of lncRNA HIF1A-AS1, which suggested that HIF1A-AS1 might be a target of TNF-α. Specifically, Kupffer cell apoptosis can be inhibited by silencing the expression of HIF1A-AS1. In addition, knockdown of HIF1A-AS1 decreased the expression of caspase 3. Finally, knockdown of the transcription factor LEF-1 can significantly reduce the expression level of HIF1A-AS1 induced by TNF-α treatment in Kupffer cells. Taken together, this study demonstrated, for the first time, as to our knowledge, that TNF-α promoted Kupffer cell apoptosis by increasing HIF1A-AS1 which targeted caspase 3 and was driven by LEF-1.

It is well known that the liver resident macrophages, Kupffer cells, are involved in the pathogenesis of liver diseases mainly by its role of antigen presenting cells and secretion of regulatory mediators.^[42]^ Growing evidences have demonstrated that TNF-α, a cytokine mainly expressed in macrophages/KCs, plays a crucial role in both liver physiological and pathological process. Of note, TNF-α is markedly up-regulated in ACLF and might play a role in the pathogenesis of human ACLF.^[43]^ Specifically, it is involved in a broad spectrum of inflammatory and immune responses, including cell differentiation, cell survival and death.^[44]^ In consistent with previous studies, we found that TNF-α showed dose-dependent cell toxicity to Kupffer cells and can effectively induce Kupffer cell apoptosis in the current study.

LncRNAs are shown to be involved in various processes, such as chromatin modification, transcription or posttranscriptional regulation, organization of protein complexes, cell–cell signaling, and allosteric regulation of proteins.^[45–47]^ In particular, lncRNAs are participated in the regulation of apoptosis. Therefore, we performed lncRNA array to screen potential lncRNAs that were involved in TNF-α-induced apoptosis of KCs. Surprisingly, we found that HIF1A-AS1 showed significant increased level in response to increased TNF-α concentration. The functions of HIFA-AS1 are poorly defined. Evidence showed that HIF1A-AS1 was up-regulated in thoracoabdominal aortic aneurysms and lncRNA HIF1A-AS1 knock-down could suppress PA-induced apoptosis of vascular smooth muscle cells in vitro.^[48]^ However, the exact functions of HIF1A-AS1 in Kupffer cells after TNF-α treatment have not been investigated. Our study, hence demonstrated, for the first time, that TNF-α regulated the expression of HIF1A-AS1 in Kupffer cells in a dose-dependent manner.

Accumulative evidence indicates that cellular apoptosis or programmed cell death is critical for the pathogenesis of TNF-dependent liver diseases.^[48]^ TNF-α induced cell apoptosis of KCs, but the potential apoptosis-related genes remained to be identified. NFKB1 regulates various substances associated with inflammation, and is activated by a wide variety of inflammatory stimuli, including lipoplysaccharides, TNF-α, and IL-1.^[49–52]^ X-linked inhibitor of apoptosis (XIAP) is the most potent inhibitor of apoptosis, which binds and inactivates effector caspsase 3 and 7 and initiator caspase 9.^[53–55]^ In addition, it induces the caspases degradation through proteasome system.^[55]^ Bad is a cytosolic proapoptotic member of Bcl-2 family members which regulate mitochondria-mediated apoptosis pathways.^[56]^ Translocation of Bad from cytosole to mitochondria causes subsequent binding with Bcl-xL, release of cytochrome c and occurrence of apoptosis.^[57]^ Therefore, we evaluated the proapototic or antiapoptotic markers by apoptosis array. As previously reported, we showed that TNF-α negatively regulated the mRNA levels of antiapoptotic genes, NFKB1, and XIAP whereas it positively regulated the mRNA levels of proapoptotic markers, Bad, caspase 3, and caspase 9. Interestingly, whether lncRNA HIF1A-AS1 are involved in regulation of these apoptosis-related genes are poorly defined. Therefore, we used siRNA transfection and TNF-α treatment to investigate the involvement of HIF1A-AS1. The current study demonstrated that inhibition of HIF1A-AS1 significantly decreased the levels of caspase 3 in KCs in response to TNF-α treatment, suggesting that caspase 3 was a target of HIF1A-AS1.

Functions of lncRNAs are extensively investigated by researchers, but how the lncRNAs are regulated remains poorly studied. Transcription factors are important for gene expressions. Hence, we evaluated whether the promoters of HIF1A-AS1 showed any binding sites for transcriptional factors with online predication method. Of note, binding sites for LEF-1 were apparently found. Thus, we detected both the mRNA and protein levels of LEF-1 in KCs treated with TNF-α. Intriguingly, no significantly differences of their levels were found after TNF-α treatment. However, knockdown of LEF-1 significantly attenuated the TNF-α-induced apoptosis. A possible explanation is that TNF-α regulates the recruitment of LEF-1 without change its expression levels to the binding sites in the promoter region of HIF1A-AS1. To test our hypothesis, we knocked down the expression of LEF-1 to minimize LEF-1's binding and detected the levels of HIF1A-AS1. Importantly, knockdown of LEF-1 significantly downregulted the levels of HIF1A-AS1 in KCs treated with TNF-α. In combination, these data suggest that TNF-α regulates HIF1A-AS1 expression through LEF-1.

In summary, we provide information, for the first time, on the mechanism of apoptosis pathways regulated by TNF-α in KCs in this study. Specifically, we show that TNF-α regulates the expression of lncRNA HIF1A-AS1 through transcription factor LEF-1. HIF1A-AS1 regulates TNF-α-induced KCs apoptosis by targeting on caspase 3. Yet, the exact mechanisms of how LEF-1 regulates HIF1A-AS1 expression are needed to be further investigated. Taken together, we provide molecular basis for clinical treatment of ACLF when the inflammatory factor TNF-α is abnormally expressed and KCs undergo apoptosis.
